# Heterogeneous Catalysis of Polyoxometalate Based Organic–Inorganic Hybrids

**DOI:** 10.3390/ma8041545

**Published:** 2015-03-31

**Authors:** Yuanhang Ren, Meiyin Wang, Xueying Chen, Bin Yue, Heyong He

**Affiliations:** Department of Chemistry and Shanghai Key Laboratory of Molecular Catalysis and Innovative Materials, Collaborative Innovation Center of Chemistry for Energy Materials, Fudan University, Shanghai 200433, China; E-Mails: YuanhangRen@fudan.edu.cn (Y.R.); 14110220018@fudan.edu.cn (M.W.); xueyingchen@fudan.edu.cn (X.C.)

**Keywords:** polyoxometalates, organic–inorganic hybrid, single crystal, heterogeneous, catalysis

## Abstract

Organic–inorganic hybrid polyoxometalate (POM) compounds are a subset of materials with unique structures and physical/chemical properties. The combination of metal-organic coordination complexes with classical POMs not only provides a powerful way to gain multifarious new compounds but also affords a new method to modify and functionalize POMs. In parallel with the many reports on the synthesis and structure of new hybrid POM compounds, the application of these compounds for heterogeneous catalysis has also attracted considerable attention. The hybrid POM compounds show noteworthy catalytic performance in acid, oxidation, and even in asymmetric catalytic reactions. This review summarizes the design and synthesis of organic–inorganic hybrid POM compounds and particularly highlights their recent progress in heterogeneous catalysis.

## 1. Introduction

Polyoxometalates (POMs) represent a diverse family of anionic molecular species that consist of early transition metal ions and oxygen atoms with high negative charge, large molecular weight, and good solubility in polar solvents. The development of POMs has received tremendous impetus, such as the publication of *Heteropoly and Isopoly Oxometalates* by Pope in 1983 [1], the critical review by Pope and Müller in 1991 [2], and a special issue of POMs by *Chemical Review* in 1998 [3,4,5,6,7]. In the new century, extensive progress has been achieved in the novelty and variety of structures and the magnetic [8,9], electronic [10,11,12], catalytic [13,14,15] and optical [14,16] properties of POMs. New areas are emerging in POM chemistry by interfacing with other fields such as biology [16], electrochemistry [17], nano-materials [18], and surface science [19]. The achievements in POM chemistry up till now have been closely related with the essential properties of POMs.

The two most unique features of POMs in catalysis application are their super acidity and excellent structural stability undergoing multi-electron redox cycles. In the past decades, several homogeneous catalytic processes based on POMs have been industrialized due to their high activity combined with low toxicity and corrosion. Considering the obvious drawbacks of homogeneous catalysis system, such as the difficulties in recycling of catalysts and purification of products, the exploration of heterogeneous POM catalysts has attracted much attention. The most commonly used route for preparation of heterogeneous POM catalysts is by loading POMs in porous materials. With this method, many heterogeneous POM-based catalysts were prepared by using various mesoporous materials with large surface areas and pore sizes as supports [20,21,22,23]. It has proven to be an effective way to obtain heterogeneous POM catalysts with a much larger surface area than bulk POMs. Although the catalysts showed noticeable activity in many catalytic reactions, they suffered aggregation and leaching of POMs due to the weak interaction between POMs and the supports. In addition, the hydrophilic property of POMs makes the catalysts inadaptable to apolar reaction systems. The amorphous nature of the catalysts also causes great difficulty in understanding their microstructure with existing techniques. As mentioned above, the development of POM-based heterogeneous catalysis calls for progress of catalysts in water tolerance, specific structure and adjustable surface polarity.

In recent years, design and synthesis of organic–inorganic hybrid POM compounds has made substantial achievement due to wide utilization of the hydrothermal synthesis technique [24,25,26]. The low solubility of organic molecules in aqueous solution can be solved under hydrothermal conditions, and the high pressure of the synthesis system is of benefit for the isolation of crystals with dynamically stable structures. Generally, the precursors for preparation of organic–inorganic hybrid POM compounds are of a three-component-system consisting of POMs, metal ions and organic ligands. The POMs act as large oxygen-enriched anionic building blocks. The metal ions are crucial structural linkers to join POMs and ligands while compensating the charge balance. The organic ligands may effectively form extended structures in various directions and dimensions through the connection of metal ions. By utilizing organic ligands with different coordination modes and configurations, a great number of organic–inorganic hybrid POM compounds have been isolated with diversified structures and topologies [25,26,27,28,29,30].

Besides their fascinating interest in crystallography, the application of organic–inorganic hybrid POMs has been explored in many areas especially in catalysis. Comparing with heterogeneous POM catalysts prepared by impregnation or the co-synthesis method, organic–inorganic hybrid POM compounds have shown several remarkable advantages. In the structures of the hybrid POM compounds, the POMs are distributed uniformly at molecular level and further firmly anchored through covalent bonds or extensive hydrogen bonds. The POMs and the metal ions in the structures are considered as potential Brønsted and Lewis acid sites, respectively. The introduction of an organic ligand increases the porosity of the structure and provides a convenient way to modify the polarity of the framework. Combining their structural clarity and water resistance, the organic–inorganic hybrid POM compounds may facilitate as a new type of POM-based catalysts having great potential in heterogeneous catalysis.

Up to now, a series of catalytic studies has been reported based on organic–inorganic hybrid POM compounds as heterogeneous catalysts. The catalytic behavior of hybrid POM compounds are notable both in activity and reusability. Due to the steric effect of the hybrid framework, the compounds show regioselectivity and even enantioselectivity. Herein the progress of heterogeneous catalysis based on organic–inorganic hybrid POM compounds is reviewed. We hope it will be beneficial in order to understand the relationship between the structure of hybrid POM compounds and their catalytic performance and to develop new hybrid POM heterogeneous catalysts.

## 2. The Design and Synthesis of Organic–Inorganic Hybrid POM Compounds

**Figure 1:**
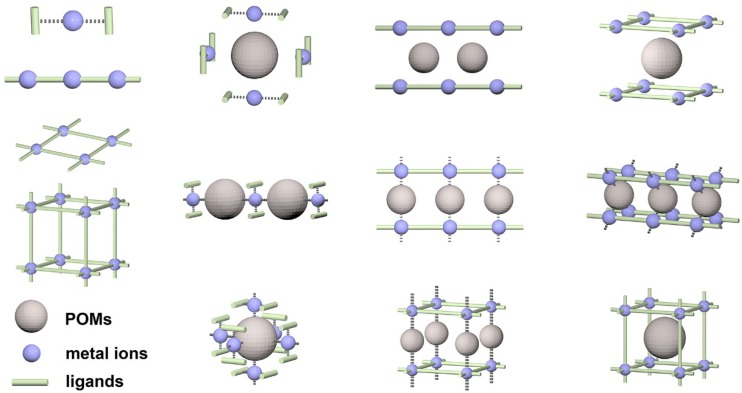
The common connection modes of metal-organic coordination complexes and the representative structures of the organic–inorganic hybrid POM compounds.

The organic–inorganic hybrid POM compounds with new topologies and high dimensional architectures are becoming an important part of POM chemistry. The most commonly studied reaction system for organic–inorganic hybrid POM compounds is the three-component-system that consists of POMs, metal ions and ligands. Hundreds of new compounds have been isolated especially by a one-pot hydrothermal reaction and some primary factors influencing the reactions have been systematically studied, including temperature, pH, molar ratio, *etc.* Commonly, the ligands coordinate with metal ions to form different dimensional structures, including zero-dimensional (0D) coordination cationic species, one-dimensional (1D) chain, two-dimensional (2D) layer, and three-dimensional (3D) framework. The coordination complexes and POM components link each other through covalent bonds or hydrogen bonds to form the organic–inorganic hybrid POM compounds. Depending on the way of connecting, the reported structures may be simply classified into typical modes as shown in Figure 1.

The POMs have multiple roles in the structure such as oxygen-enriched ligands, inorganic templates, and structural nodes. The widely used organic ligands are nitrogenous heterocyclic molecules and carboxylates. The chelate ligands may efficiently stabilize the metal ions to form 0D coordination cations and the linear ligands show a trend to form high dimensional extended structures. The formation of organic–inorganic hybrid POM compounds is often described as a self-assemble process because of the intricate behavior of POMs, metal ions, and ligands during crystallization which causes a lot of difficulty in predicting the structure and composition of the products. The rational design and synthesis of organic–inorganic hybrid POM compounds remains a big challenge.

**Figure 2:**
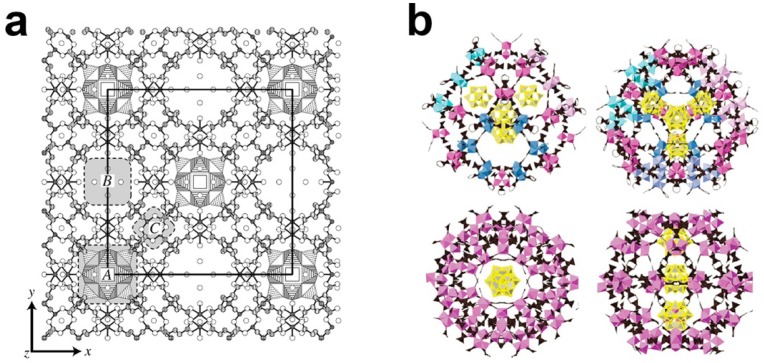
(**a**) The HKUST-1 framework and Keggin anions are represented by ball-and-stick and polyhedral models, respectively. Reproduced with permission from Elsevier, 2003 [31]; (**b**) Proposed crystal structure of MIL-101/PTA. Reproduced with permission from America Chemical Society (ACS), 2012 [38].

Instead of the self-assemble process which has much uncertainty, synthesis of organic–inorganic hybrid POM compounds by incorporating POMs into prefabricated MOFs (metal-organic frameworks) is also an interesting method. One of the representative compounds is a Keggin-type polyoxoanion incorporated HKUST-1 reported by Yamase in 2003 [31]. As shown in Figure 2a, the big pores in the framework of HKUST-1 are occupied by Keggin polyoxoanions due to perfect matching in size and symmetry. This method was explored for the synthesis of Materials of Institute Lavoisier (MIL) [32,33,34,35] series of porous materials with large pore size and opening windows which allow the incorporation of POMs within the pores (Figure 2b). The studies showed that POMs could be encapsulated in the pores of MIL materials by impregnation, one-pot synthesis, or microwave treatment [36,37]. The precise positions of the POMs in MIL were difficult to determine by single X-ray diffraction but the POMs were observed to be firmly entrapped inside the pores as proven by leaching tests [38]. The high loading of POMs and significant porosity in the porous structure of MIL facilitate catalytic reactions.

## 3. Acid Catalysis

**Figure 3:**
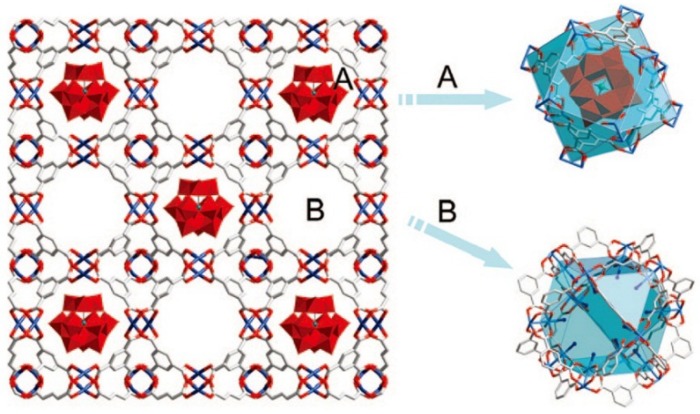
Polyhedral and ball-and-stick representation of NENU**-**3 Colour code: Blue, Cu; red, O; gray, C. Reproduced with permission from ACS, 2009 [39].

In 2009, Liu and co-workers reported the NENU (North East Normal University) series of organic–inorganic hybrid POM compounds by the one-pot hydrothermal reaction of Cu ions, BTC (1,3,5-benzencarbonxylate) and Keggin-type polyoxoanions (H_4_SiW_12_O_40_·*n*H_2_O, H_4_SiMo_12_O_40_·*n*H_2_O, H_4_GeW_12_O_40_·*n*H_2_O, H_3_AsMo_12_O_40_·*n*H_2_O, H_3_PW_12_O_40_·*n*H_2_O, or H_3_PMo_12_O_40_·*n*H_2_O) [39]. In the structures of the NENU compounds, Cu ions and BTC ligands form a three dimensional rigid framework which represents an identical structural feature with the famous HKUST-1 MOFs. There are two types of cavities, A and B, in the Cu-BTC framework with diameters of 13 and 11 Å and sizes of accessible windows of 11 and 9.3 Å, respectively. As shown in Figure 3, cavity A is occupied by Keggin polyoxoanions and cavity B is occupied by (CH_3_)_4_N^+^ cations and water molecules. It may be clearly observed from the crystal structure of these compounds that the hydrophilic Keggin polyoxoanions are dispersed uniformly in the hydrophobic Cu-BTC matrix. Considering the window size of cavity A is smaller than the diameter of Keggin polyoxoanion, the Keggin unit is blocked inside the cavity which prohibits leaching of Keggin polyoxoanions during catalytic reactions. The [PW_12_O_40_]^3−^ polyoxoanion contained compound NENU-3 may form a dehydrated product NENU-3a after heating at 473 K without collapse of the structure. The acid-base titration of NENU-3 indicates that the amount of acidic protons is 1.8 × 10^−4^ mol·g^−1^ which corresponds to one proton per [PW_12_O_40_]^3−^ polyoxoanion, and the acidic proton content of NENU-3a is 5.7 × 10^−4^ mol·g^−1^ which corresponds to three protons per [PW_12_O_40_]^3−^ polyoxoanion. Hydrolysis of ester in water was chosen to test the acid catalytic properties of the compounds. NENU-3a showed the highest catalytic activity for hydrolysis of ethyl acetate in excess of water. The conversion of ethyl acetate was >95% after 7 h reaction, which was far superior to the most commonly used inorganic and organic acids, such as H_2_SO_4_, SO^4−^/ZrO_2_, H-ZSM-5, Nb_2_O_5_ and Nafion-H. It should be noted that HKUST-1 showed no catalytic activity and the simple mixture of HKUST-1 and H_3_PW_12_O_40_ gave low conversion in the same reaction. The results indicated that the activity derived from the [PW_12_O_40_]^3−^ polyoxoanions in NENU-3a and the hydrophobic Cu-BTC framework improved the adsorption ability of the hydrophilic polyoxoanion with weak polar esters. The activities of NENU-3a in the hydrolysis of various esters were also studied to investigate the influence of the size of the reactants. Methyl acetate and ethyl acetate showed similar conversions (~64%) after 5 h reaction, but the conversions decreased dramatically when bigger reactants were introduced. Conversion of 4-methyl-phenyl propionate was below 1% after 24 h reaction. The catalyst could be recycled fifteen times without losing its reactivity. The solution became inactive after the catalyst was filtered and no leaching of polyoxoanion was observed from a Ultraviolet–Visible spectroscopy (UV-Vis) study. These features indicated that NENU-3a operated as a heterogeneous catalyst in the reactions.

The catalytic activity of NENU-1a was further assessed by the dehydration of methanol to dimethyl ether [40]. A series of NENU-1a were isolated by the hydrothermal reaction of copper salt, BTC, and [SiW_12_O_40_]^4−^ polyoxoanion. Crystals with different particle sizes of 23, 105, and 450 μm were isolated by controlling the concentration of the reactants and reaction temperature. The activity of NENU-1a as heterogeneous catalyst was determined in a fixed-bed reactor. The conversions of methanol over NENU-1a (105 μm) increased from 58% at 513 K to 85% at 773 K which were much higher than over γ-Al_2_O_3_ or γ-Al_2_O_3_ supported [SiW_12_O_40_]^4−^ catalyst. The conversion over pure Cu-BTC framework without [SiW_12_O_40_]^4−^ was only 9.1% at 513 K when a rapid deactivation of the catalyst occurred due to the collapse of the crystal structure at higher temperature. This result indicated that the [SiW_12_O_40_]^4−^ polyoxoanions in the Cu-BTC framework enhanced the thermal stability of the Cu-BTC framework dramatically. When an excess amount of water was introduced, the conversion of methanol over NENU-1a only decreased slightly, demonstrating the high resistance against water of Brønsted acid sites of the [SiW_12_O_40_]^4−^ polyoxoanion. Following the change of particle size of the NENU-1a crystal, the mass transfer effect could be observed. The NENU-1a catalysts with smaller particle sizes of 23 and 105 μm gave a similar conversion of methanol which was higher than that of the catalyst with particle size of 450 μm.

Moreover, through modifying the preparation method by mixing the reactants at room temperature and quenching in liquid nitrogen, nanocrystals of [PW_12_O_40_]^3−^ containing HKUST-1 compound with particle size of 50 nm were obtained [41]. The activity found for esterification of acetic acid and 1-propanol showed that a catalyst with a smaller particle size significantly affected the catalytic activity. Under the same reaction condition, conversion over 50 nm-sized nanocrystals was three times higher than that of the 20 μm-sized microcrystals.

**Figure 4:**
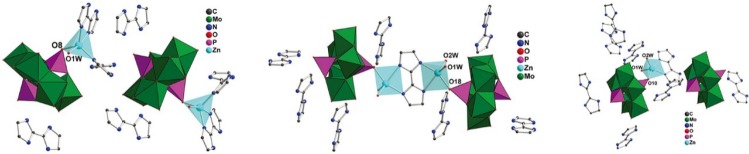
Combined polyhedral and ball-and-stick representation for the three hybrid compounds based on [P_2_Mo_5_O_23_]^6−^ and Zn-H_2_biim coordination complexes. Reproduced with permission from RSC, 2014 [42].

In 2014, Zhu and co-workers reported three supramolecular POM compounds by the hydrothermal reaction of Na_2_MoO_4_·2H_2_O, H_3_PO_4_, H_2_biim (2,2'-biimidazole), and ZnSO_4_·7H_2_O [42]. In these compounds, Na_2_MoO_4_ and H_3_PO_4_ form Strandberg-type polyoxoanions [P_2_Mo_5_O_23_]^6−^ modified by Zn-H_2_biim coordination complexes and further packed into a three dimensional structure through hydrogen bonds, as shown in Figure 4. The three compounds were explored as acid catalysts for catalytic ketalization of cyclohexanone with glycol, giving high yields of cyclohexanone ethylene ketal (86%, 99%, and 95%). In contrast, the catalytic activity over ZnSO_4_ or [P_2_Mo_5_O_23_]^6−^ polyoxoanions was much lower than the hybrid compounds, indicating that the catalytic activity contributed to the synergetic effect of the components.

**Figure 5:**
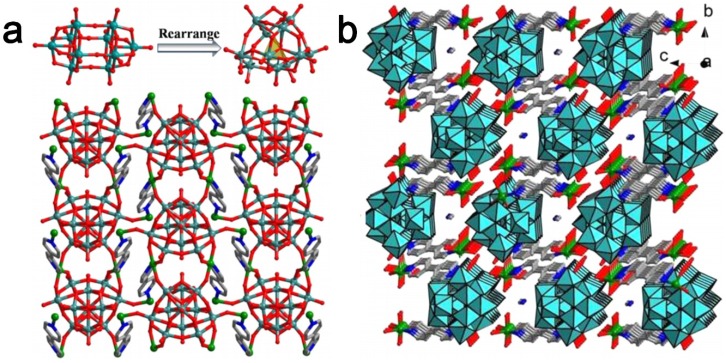
(**a**) The rearrangement of [W_10_O_32_]^4−^ and the 2D framework constructed by [H_2_W_11_O_38_]^8−^ polyoxoanions and copper complexes; (**b**) Stacking pattern of the compound and the 1D channels viewing along the *a* axis. Reproduced with permission from ACS, 2014 [43].

Recently, Niu and coworkers reported an organic–inorganic hybrid POM compound, {[Cu_2_(bpy)(H_2_O)_5.5_]_2_[H_2_W_11_O_38_]·3H_2_O·0.5CH_3_CN} (bpy = 4,4'-bipyridine), by reaction of [(C_4_H_9_)_4_N]_4_W_10_O_32_, Cu(NO_3_)_2_·6H_2_O and 4,4'-bipyridine in a mixture of water and acetonitrile [43]. The precursor [W_10_O_32_]^4−^ polyoxoanions rearranged into [H_2_W_11_O_38_]^8−^ building blocks which were further connected into 2D frameworks by copper complexes (Figure 5). The compound was used as a heterogeneous acid catalyst for cyanosilylation of aromatic aldehydes and cyanotrimethylsilane in CH_3_CN at room temperature. The yield of 2-phenyl-2-(trimethylsilyloxy)acetonitrile reached about 98.1% by reaction of benzaldehyde with cyanotrimethylsilane. The yield decreased correspondingly when the molecular size of the aromatic aldehydes increased. The catalytic cyanosilylation reaction in the presence of 3-formyl-1-phenylene-(3,5-di-tert-butylbenzoate) gave less than 52.4% conversion under the same reaction conditions. The adsorption experiments indicated that the 3-formyl-1-phenylene-(3,5-di-tert-butylbenzoate) was too large to be adsorbed in the channels of the compound, which indicated that the cyanosilylation reaction occurred in the channels instead of on the external surfaces.

## 4. Oxidation Catalysis

### 4.1. Oxidation of Ethylbenzene

**Figure 6:**
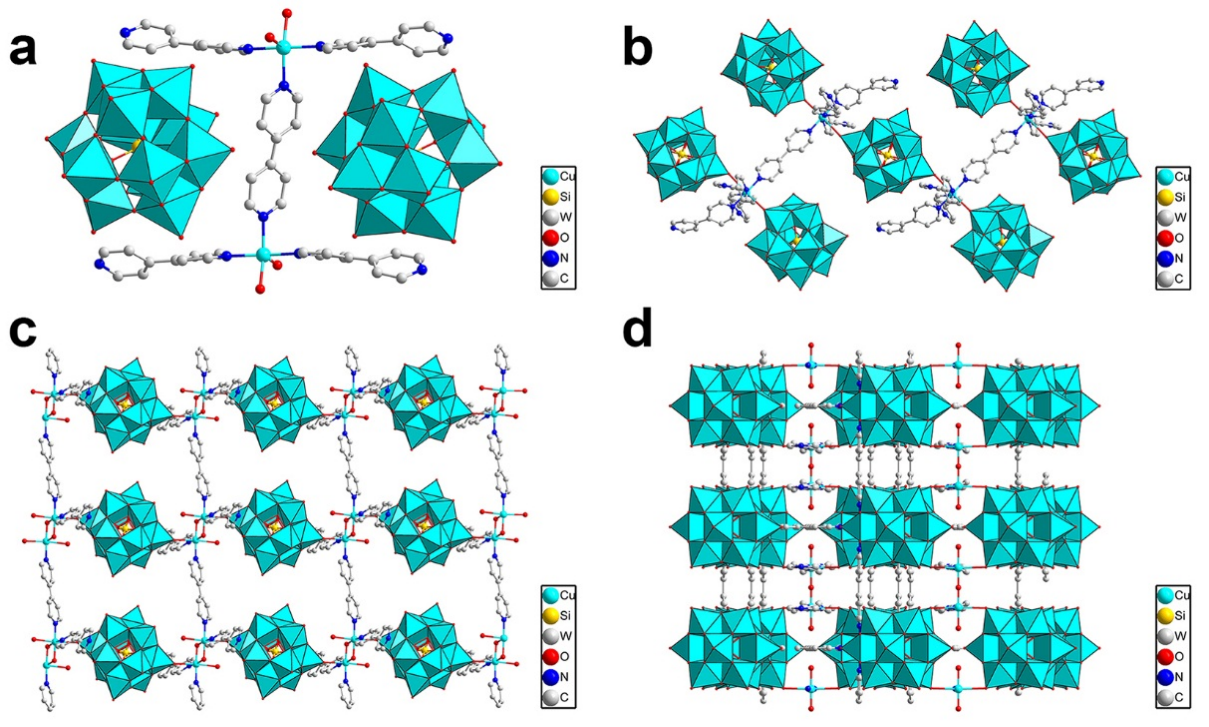
(**a**–**d**) Polyhedral and ball-and-stick representation for the compounds **1**–**4** based on [SiW_12_O_40_]^4−^ polyoxoanion.

Long *et al.* reported the synthesis of four organic–inorganic hybrid POM compounds, {[Cu_2_(4,4'-bpy)(4,4'-Hbpy)_4_(H_2_O)_4_](SiW_12_O_40_)_2_(H_2_O)_4_}*_n_* (**1**), {[Cu_2_(4,4'-bpy)(4,4'-Hbpy)_6_(SiW_12_O_40_)_3_](4,4'-Hbpy)_2_(H_2_O)_7_}*_n_* (**2**), {[Cu_2_(μ_2_-H_2_O)_2_(4,4'-bpy)_3_(SiW_12_O_40_)](H_2_O)_6_}*_n_* (**3**), {[Cu_2_(μ_2_-OH)(4,4'-bpy)_3_(SiW_12_O_40_)(H_2_O)][Cu_2_(μ_2_-O)(4,4'-bpy)_4_(H_2_O)_2_]_0.5_(H_2_O)_3_}*_n_* (**4**), by reaction of Keggin-type [SiW_12_O_40_]^4−^ polyoxoanion, Cu ion, and 4,4'-bpy under hydrothermal conditions [44]. The Cu ions and 4,4'-bpy molecules are connected to form four different types of dinuclear copper coordination cations under different pH conditions. The POM units locate between the coordination cations to form four compounds with 3D frameworks through hydrogen bonds as shown in Figure 6.

The catalytic activities for oxidation of ethylbenzene with TBHP (tert-butyl hydroperoxide) as oxidant over the four compounds were investigated as shown in Table 1. The catalytic results showed that the products contained acetophenone, 1-phenylethanol, and benzaldehyde, and the main product for all four compounds was acetophenone. The best compound gave a conversion of 51.1% and acetophenone selectivity of 85.2%. The leaching test and recycling experiments indicated that the reaction is a heterogeneous process. Since the pore size is smaller than the reactant for the reaction, the porosity of the compounds should not be the key factor for their catalytic activity. The catalytic activities showed a significant relationship with acidity instead of with the amount of Cu^II^ ions. The sequence of catalytic activity is opposite to the amount of protonated 4,4'-bpy molecules in the four compounds.

**Figure 7:**
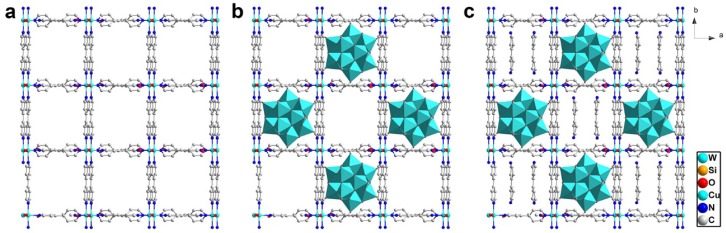
(**a**) The 2D layer of [Cu(4,4'-bpy)_2_(H_2_O)_2_]*_n_*^2*n*+^ in compounds **5**–**8**; (**b**) Polyhedral and ball-and-stick representation for compounds **5**–**7**; (**c**) Polyhedral and ball-and-stick representation for compound **8**.

By selecting Keggin-type polyoxoanions and the four-connected 2D layer of [Cu(4,4'-bpy)_2_(H_2_O)_2_]*_n_*^2*n*+^, Long and co-workers further isolated four hybrid POM compounds **5**–**8**, {[Cu_2_(4,4'-bpy)_4_(H_2_O)_4_](SiW_12_O_40_)(H_2_O)_18_}*_n_* (**5**), {[Cu_2_(4,4'-bpy)_4_(H_2_O)_4_](PW_12_O_40_)(H_2_O)_18_}*_n_* (**6**), {[Cu_2_(4,4'-bpy)_4_(H_2_O)_4_](PMo_12_O_40_)(H_2_O)_18_}*_n_* (**7**) and {[Cu_2_(4,4'-bpy)_4_(H_2_O)_4_](SiW_12_O_40_)(4,4'-bpy)_2_(H_2_O)_4_}*_n_* (**8**) [45,46]. As shown in Figure 7b, compounds **5**–**7** are isostructural with different Keggin-type POMs ([SiW_12_O_40_]^4−^ for **5**, [PW_12_O_40_]^3−^ for **6** and [PMo_12_O_40_]^3−^ for **7** and the same voids in a 2D layer connected by Cu ions and 4,4'-bpy molecules. Compound **8** has the same framework as those for compounds **5**–**7**, but the voids in compound **8** are partially occupied by 4,4'-bpy guest molecules (Figure 7c). The high structural similarity of compounds **5**–**8** offers an ideal model to understand the influence of composition and porosity on their catalytic activity.

**Table 1:** Catalytic results for oxidation of ethylbenzene over compounds **1**–**8**.

Compound	Component		Conversion (%)	Selectivity to Acetophenone (%)
	POMs	Metal Ion		
**1**	SiW_12_	Cu^II^	27.6	76.0
**2**	SiW_12_	Cu^II^	22.4	75.9
**3**	SiW_12_	Cu^II^	51.1	85.2
**4**	SiW_12_	Cu^II^	44.3	82.0
**5**	SiW_12_	Cu^II^	56.8	88.1
**6**	PW_12_	Cu^II^	44.5	82.2
**7**	PMo_12_	Cu^II^	45.1	86.5
**8**	SiW_12_	Cu^II^	37.9	80.1

The catalytic results for oxidation of ethylbenzene over compounds **5**–**8** are listed in Table 1. The influences of solvent, reaction temperature, reaction time, amount of oxidant were systematically studied. The optimized condition study of the reaction showed that the highest yield of acetophenone was achieved with a reaction temperature of 313–343 K, reaction time of 6–12 h, TBHP/ethylbenzene ratio of 2/1, and using acetonitrile as a solvent. The best compound **5** gave a conversion of 56.8% and acetophenone selectivity of 88.1%.

The high structural similarity of the four compounds **5**–**8** made it convenient to understand the effect of the hybrid POM compounds on their catalytic activity. The catalytic activity of the three isostructural compounds **5**–**7** containing different Keggin anions was in the order of {SiW_12_} > {PMo_12_} > {PW_12_}, which indicated that activity of the hybrid compounds decreased with the increase of acidity of the POM component. It should be noted that the acidity of Keggin units did not show a strong effect on their catalytic activities, the difference in conversion was around 10% and the selectivities were nearly the same. The {SiW_12_}-based compound **5** with water as guest molecules in the framework showed the highest activity.

When the {SiW_12_}-based compound **5** was replaced by compound **8** with the same framework but with the guest molecules of water replaced by 4,4'-bpy, the catalytic activity clearly decreased. The guest molecules 4,4'-bpy in the structure of compound **8** could be slowly removed from the porous framework by soaking the crystals in acetonitrile. The oxidation of 1-phenylethanol to acetophenone was chosen as a model reaction to study the effect of guest molecules in the pore of the structure. In the initial stage of the reaction, compound **8** with guest molecules 4,4'-bpy in the framework gave low activity. When the 4,4'-bpy molecules were removed during the reaction, the activity of compound **8** increased significantly. It could be concluded that the oxidation reaction occurred in the pore of the framework.

### 4.2. Oxidation of Styrene

The epoxidation of styrene is another model reaction used to study the catalytic activity of POM-based hybrid compounds. Long *et al.* reported the catalytic results of epoxidation of styrene over four {SiW_12_}-based compounds **1**–**4** with TBHP as oxidant. The compounds showed high catalytic activity but the main product was benzaldehyde [44]. The distribution of the products indicated that the reaction was acid sensitive. The protonated 4,4'-bpy ligand in these compounds led to the ring opening of the epoxide.

We have synthesized a series of hybrid POM compounds with Keggin-type anions, Cu or Ag ions, and 4,4'-bpy and 2,2'-bpy or 4,4'-bpy and phen (phen = phenanthroline) as mixed ligands, [Ag_4_(4,4'-bpy)_3_(2,2'-bpy)_2_][SiW_12_O_40_]·2H_2_O (**9**), [Cu(4,4'-bpy)(2,2'-bpy)_2_]_2_[SiW_12_O_40_]·4H_2_O (**10**), [Cu(4,4'-bpy)(phen)]_2_[H_3_O]_2_[SiW_12_O_40_]·8H_2_O (**11**) and [Cu(4,4'-bpy-Cl)(phen)]_2_[H_3_O][PW_12_O_40_]·H_2_O (**12**) [47]. The four compounds have a bulk 3D H-bond network without an open framework. Table 2 shows that the conversions of the Cu-based compounds **10**–**12** are over 80%, which are significant higher than the Ag-based compound **9** (15.3%). By comparing the catalytic activity of the two compounds **11** and **12** with high structural similarity, one may find that the {SiW_12_}-based compound is slightly higher than the {PW_12_}-based compound.

**Figure 8:**
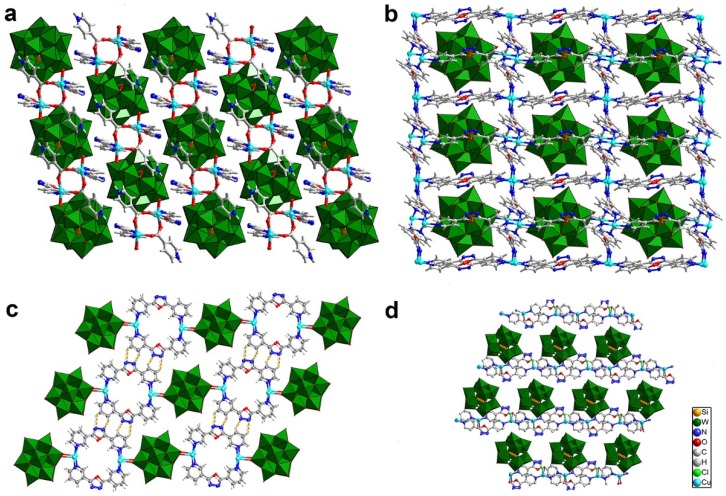
(**a**–**d**) Polyhedral and ball-and-stick representation for compounds **13**–**16**.

A series of {SiW_12_}-based compounds, [Cu_2_(C_5_H_5_NCOO)_2_(4-bpo)_2_(H_2_O)_2_]SiW_12_O_40_·H_2_O (**13**), [Cu_4_(4-bpo)_6_]SiW_12_O_40_·3H_2_O (**14**), [Cu_4_(3-bpo)_4_]SiW_12_O_40_·3H_2_O (**15**) and [Cu(4-bpo)(H_2_O)][Cu_2_(μ_2_-Cl)(4-bpo)_2_(H_2_O)][SiW_12_O_40_][N(CH_3_)_4_]_2_·H_2_O (**16**), were prepared by reaction of [SiW_12_O_40_]^4−^, copper salt and 3/4-bpo (2,5-bis(3/4-pyridyl)-1,3,4-oxadiazole) under hydrothermal conditions [48,49]. Compound **13** consists of [SiW_12_O_40_]^4−^ polyoxoanions and a type of [Cu_2_(C_5_H_5_NCOO)_2_(4-bpo)_2_(H_2_O)_2_]*_n_*^4*n*+^ 1D chains as shown in Figure 8a. In compound **14**, the Cu ions and the 4-bpo molecules connect into a 2D layer and the [SiW_12_O_40_]^4−^ polyoxoanions locate in the cages of the 2D layer (Figure 8b). The Cu ions in compound **15** are joined into binuclear coordination cations by 3-bpo molecules and the binuclear cations are further connected into a 3D hydrogen bonding network by [SiW_12_O_40_]^4−^ polyoxoanions as shown in Figure 8c. Compound **16** contains two types of 1D chains, [Cu(4-bpo)(H_2_O)]*_n_* and [Cu_2_(μ_2_-Cl)(4-bpo)_2_(H_2_O)]*_n_* and the [SiW_12_O_40_]^4−^ polyoxoanions occupy the cavities between the chains to form a 3D hydrogen bonding structure (Figure 8d).

The catalytic activity for epoxidation of styrene over compounds **13**–**16** is shown in Table 2. The highest conversion over {SiW_12_}-based compound **14** reaches 93.8%. However, most of the {SiW_12_}-based compounds show no obvious selectivity between styrene epoxide and other deep oxidation products such as benzaldehyde and benzoic acid.

**Figure 9:**
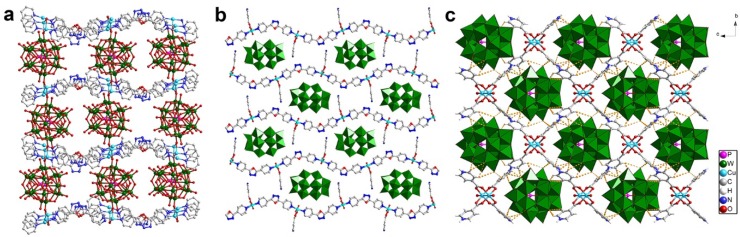
(**a**) Polyhedral and ball-and-stick representation for compound **17**; (**b**) Space-filling and ball-and-stick representation for compound **18**; (**c**) Ball-and-stick representation for compound **19**.

Beside the Keggin-based compounds, we synthesized several Dawson-based hybrid compounds, [Cu(4-bpo)]_4_[P_2_W_18_O_62_][N(CH_3_)_4_]_2_·6H_2_O (**17**), [Cu_2_(μ_2_-OH)(4-bpo)_2_(Hina)(H_2_O)_2_]_2_[P_2_W_18_O_62_]·4H_2_O (**18**) and [Cu_2_(Hina)_4_(H_2_O)_2_][H_2_P_2_W_18_O_62_](Hina)·11H_2_O (**19**), (ina = isonicotinic acid) [49]. Compound **17** shows a 3D rigid framework connected by [P_2_W_18_O_62_]^6−^ polyoxoanions, Cu^I^ ions and 4-bpo molecules. As shown in Figure 9a, the [P_2_W_18_O_62_]^6−^ polyoxoanions locate between the Cu^I^-4-bpo layers. In compound **18**, the Cu^II^ ions are connected into a 1D chain by the ligands and the [P_2_W_18_O_62_]^5−^ polyoxoanions locate in the voids between the adjacent chains (Figure 9b). The Cu^II^ ions are joined into a type of binuclear saddle units, the units packing with the [H_2_P_2_W_18_O_62_]^4−^ polyoxoanions through hydrogen bonding form the 3D structure of compound **19** as shown in Figure 9c. 

**Table 2:** The catalytic results for epoxidation of styrene over compounds **9**–**19**.

Compound	Component		Conversion (%)	Selec. (%) *^a^*	Selec. (%) *^b^*	Selec. (%) *^c^*
	POMs	Metal Ion				
**9**	SiW_12_	Ag^I^	15.3	23.2	71.1	5.7
**10**	SiW_12_	Cu^II^	83.9	15.8	65.3	18.9
**11**	SiW_12_	Cu^I^	91.9	62.2	25.2	12.6
**12**	PW_12_	Cu^I^	85.1	57.2	29.3	13.5
**13**	SiW_12_	Cu^II^	90.6	44.7	33.7	26.1
**14**	SiW_12_	Cu^I^	93.8	35.6	35.4	29.0
**15**	SiW_12_	Cu^I^	56.0	49.5	43.1	7.4
**16**	SiW_12_	Cu^I^	48.2	60.2	34.7	5.1
**17**	P_2_W_18_	Cu^I^	73.2	69.4	26.4	4.2
**18**	P_2_W_18_	Cu^II^	76.7	33.0	61.4	5.6
**19**	P_2_W_18_	Cu^II^	70.3	22.9	71.0	6.1

*^a^* Styrene oxide selectivity (%). *^b^* Benzaldehyde selectivity (%). *^c^* Others (benzoic acid, *etc.*) selectivity (%).

As shown in Table 2, the hybrid Dawson compounds **17**–**19** display high activity in the epoxidation of styrene, and conversions are over 70%. The compounds show completely different behavior from that of [P_2_W_18_O_62_]^6−^ although the latter proceeds in fact in a homogeneous system. These results imply that the catalytic activity of the Dawson-based hybrid compounds comes from their entire structures instead of any individual components. The differences in the amount of Cu and ligands does not play a key role in their activity. The effect of the POM type is particularly obvious between examples **16** and **17** where both contain Cu^I^ centers in similar coordination geometries with similar 2D compact hybrid layers but have different types of polyoxoanions located between the layers. The {P_2_W_18_}-based compound **17** shows higher activity than the {SiW_12_}-based compound **16**.

Besides the well-known saturated Keggin or Dawson polyoxoanions, hybrid compounds of other types of polyoxoanions, such as the substituted-Keggin, capped-Keggin, and classical Anderson also showed activity in the oxidation of styrene. In 2014, Lu and co-workers reported the organic–inorganic hybrid POM compound based on Cu, 4,4'-bpy and [PMo_11_NiO_39_]^5−^ polyoxoanions [50]. The Cu ions and 4,4'-bpy ligands connect into 1D chains and the Ni mono-substituted [PMo_11_NiO_39_]^5−^ polyoxoanions are connected into the chains through Ni–N bonds. In the oxidation of styrene with TBHP as oxidant, the conversion of styrene reached 87.2% and the selectivity to benzaldehyde was about 80% after 60 h reaction.

**Figure 10:**
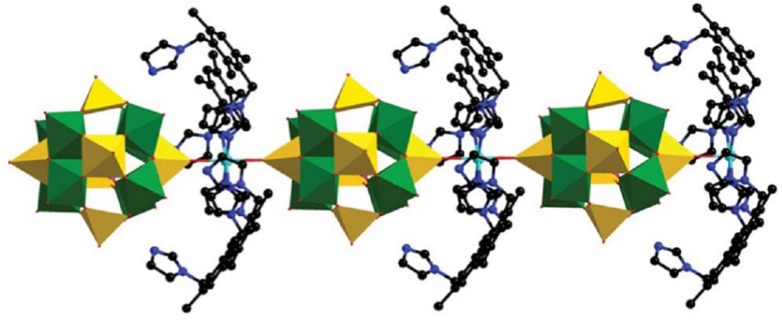
Polyhedral and ball-and-stick representation for the hybrid compound [Ni(m-bitmb)_4_][H_5_PMo^VI^_8_V^IV^_4_O_40_(V^IV^O)_2_]. Reproduced with permission from RSC, 2012 [51].

Xu *et al.* reported an organic–inorganic hybrid POM compound obtained by hydrothermal reaction of Na_2_MoO_4_, NH_4_VO_3_, Ni(OAc)_2_, H_3_PO_4_, and m-bitmb (1,3-bis(1-imidazol-1-ylmethyl)-2,4,6-trimethylbenzene) [51]. Eight Mo atoms and four V atoms are connected into a mixed-addenda Keggin-type {PMo_8_V_4_} which is further coordinated by two VO_2_ units to form a bi-capped [H_5_PMo^VI^_8_V^IV^_4_O_40_(V^IV^O)_2_]^2−^ polyoxoanion as shown in Figure 10. The bi-capped polyoxoanions and the [Ni(m-bitmb)_4_]^2+^ moieties are connected alternatively to form a 1D chain. The oxidation of styrene by H_2_O_2_ was studied over the compound as catalyst. The conversion of styrene reached 90% and the selectivities were 69.7%, 26.4%, and 3.9% for benzaldehyde, benzoic acid and epoxide, respectively.

Two organic–inorganic hybrid POM compounds based on Anderson-type polyoxoanion [TeMo_6_O_24_]^6−^ or [TeW_6_O_24_]^6−^ were prepared by Ali *et al.* [52]. The results for oxidation of styrene with TBHP as oxidant over the two compounds showed that the main products were styrene epoxide and diols. The conversion of styrene over the [TeMo_6_O_24_]^6−^ containing compound was 89.8% and the selectivity to styrene epoxide was 59.2% after 12 h.

### 4.3. Oxidation of Alkane

**Figure 11:**
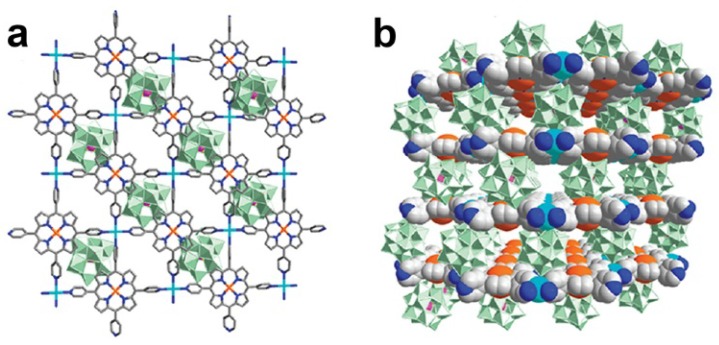
(**a**) Arrangement of a single layer of [Cd(DMF)_2_Mn^III^(DMF)_2_TPyP]*_n_*^3*n*+^ and a layer of [PW_12_O_40_]^3−^ polyoxoanions, as viewed along the *c* axis; (**b**) Packing mode of [Cd(DMF)_2_Mn^III^(DMF)_2_TPyP]*_n_*^3*n*+^ layers and [PW_12_O_40_]^3−^ polyoxoanions. Reproduced with permission from ACS, 2012 [53].

Wu and co-workers isolated an organic–inorganic hybrid POM compound constructed by Mn^III^-porphyrin, Cd ion and [PW_12_O_40_]^3−^ polyoxoanion under ambient condition [53]. The Mn^III^-TPyP (TPyP = tetrapyridylporphyrin) are connected by Cd ions to form a 2D [Cd(DMF)_2_Mn^III^(DMF)_2_TPyP]*_n_*^3*n*+^ (DMF = *N*,*N*-dimethylformamide) layer along the *ab* plane (Figure 11a). As shown in Figure 11b, the Keggin-type [PW_12_O_40_]^3−^ polyoxoanions only occupy half of the cavities between the adjacent layers as counter ions. As a result, an accessible porous structure is formed with an opening window of 5.36 Å × 12.44 Å in the compound.

The compound showed notable catalytic activity for the oxidation of alkylbenzenes to phenyl ketones. For the oxidation of ethylbenzene, the yield of acetophenone was 92.7% in the first run and only a slight decrease was observed during the recycling runs. For comparison, the catalytic activity of oxidation of ethylbenzene over Mn^III^-TPyP or [PW_12_O_40_]^3−^ polyoxoanion was also tested. The [PW_12_O_40_]^3−^ polyoxoanion showed null activity and the yield over Mn^III^-TPyP was 73.6% but decreased dramatically to 2.6% in the second run. Thus showing that the activity is derived from the Mn^III^-TPyP and the [PW_12_O_40_]^3−^ polyoxoanion which prevent rapid deactivation by holding the Mn^III^-TPyP components in a robust framework instead of leaching and formation of inactive metalloporphyrin dimers in solution.

When alkylbenzenes with a large molecular size were applied as substrates, the yield of phenyl ketones decreased significantly. The results of the liquid phase adsorption experiments showed that the ethylbenzene molecules could enter into the framework of the compound, but the larger alkylbenzene molecules could not be detected in the pores due to steric hindrance. The high uptake amount of ethylbenzene in the framework (13.2 wt%, determined by Gas Chromatography-Mass Spectroscopy (GC-MS) indicated that the internal surface of the organic–inorganic hybrid compound displayed considerable hydrophobicity even with the existence of polar polyoxoanions.

### 4.4. Oxidative Desulfurization

Nowadays, environmental regulations have become much stricter for limiting sulfur content in diesel fuel because diesel powered vehicles contribute greatly to urban air pollution. The widely used hydrodesulfurization (HDS) technique is highly efficient in removing thiols, sulfide, and disulfide from fuels, however it is less effective for the refractory sulfur-containing aromatic hydrocarbon compounds, such as benzothiophene (BT), dibenzothiophene (DBT), 4,6-dimethyldibenzothiophene (4,6-DMDBT) and other derivatives. To meet the requirement in fuel quality control, the oxidative desulfurization (ODS) technique has of necessity attracted much attention in recent years. In contrast to the required high temperature and high pressure HDS process, the ODS technique can oxidize the refractory sulfur compounds under mild conditions which is considered as complementary to the ultra-deep desulfurization process. So far, POM-based catalysts have been widely studied in the ODS process because of their high activity in conversion of DBT to sulfone.

Liu and co-workers reported a facile synthesis route to prepare the [PMo_10_V_2_O_40_]^5−^ containing NENU-9 nanocrystals with various particle sizes [54]. The nanocrystals of NENU-9 were used as a heterogeneous catalyst for oxidation of DBT with molecular oxygen as oxidant. After 60 min reaction over NENU-9 nanocrystals with average size of 550 nm, 90% of DBT was converted to DBTO_2_, and complete conversion was achieved after 90 min. The small size of the nanoparticle significantly improved the mass transfer during the catalytic process as the conversion over the NENU-9 with average size of 300 μm was only 41% under the same conditions.

The amphipathic surface of NENU-9 is also crucial to the interaction between hydrophobic DBT and the hydrophilic polyoxoanions components. In contrast, the conversion over hydrophilic [PMo_10_V_2_O_40_]^5−^ polyoxoanions was only 2% due to its weak interaction with the hydrophobic DBT.

**Figure 12:**
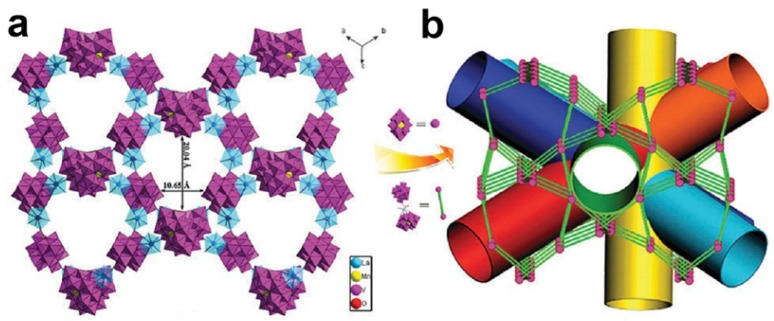
(**a**) Polyhedral representation for the frameworks constructed by lanthanide ion and [MnV_13_O_38_]^7−^ polyoxoanion in compounds H[La(H_2_O)_4_]_2_[MnV_13_O_38_]·9NMP·17H_2_O and H[Ce(H_2_O)_4_]_2_[MnV_13_O_38_]·9NMP·17H_2_O; (**b**) The representation of the multi-directional channel system in the compounds. Reproduced with permission from RSC, 2014 [55].

Desulfurization may also be catalyzed by hybrid POM compounds based on other types of polyoxoanions. In 2014, Wang and co-workers reported a series of 3D frameworks, H[La(H_2_O)_4_]_2_[MnV_13_O_38_]·9NMP·17H_2_O and H[Ce(H_2_O)_4_]_2_[MnV_13_O_38_]·9NMP·17H_2_O (NMP = *N*-methyl-2-pyrrolidone), by reaction of lanthanide ion (La and Ce), NMP and [MnV_13_O_38_]^7−^ polyoxoanion [55]. In the structure of the compounds, the [MnV_13_O_38_]^7−^ polyoxoanions connect with lanthanide ions to form a 3D porous framework which contains a multi-directional channel system along six different directions as shown in Figure 12.

The framework constructed by Ce ions and [MnV_13_O_38_]^7−^ can catalyze the oxidation of thiophene sulfides to sulfones with tert-butyl hydroperoxide as the oxidant. The maximum conversions were 98.8% achieved after 4 h for BT, 99.5% after 3 h for DBT and 99.1% after 6 h for 4,6-DMDBT. Moreover, the three substrates were oxidized into the corresponding sulfones with 100% selectivity. The framework showed good stability during the catalytic process and the conversion over the recovered catalyst in successive runs only decreased slightly.

Liu *et al.* reported the oxidative desulfurization over [PW_12_O_40_]^3−^ encapsulated MIL-101(Cr) catalyst namely PTA@MIL-101(Cr) [56]. The PTA@MIL-101(Cr) catalyst was prepared through a one-pot hydrothermal reaction by introducing [PW_12_O_40_]^3−^ polyoxoanions as reactant during the synthesis of MIL-101(Cr). The loading amount of [PW_12_O_40_]^3−^ polyoxoanions can reach 50 wt% and the lattice of MIL-101(Cr) only changes slightly. The catalytic activity of the PTA@MIL-101(Cr) was tested using the model oil consisting of sulfur compounds and n-heptane with H_2_O_2_ as oxidant (H_2_O_2_/S = 50). The conversion of DBT was very low by using 50 wt% PTA@MIL-101(Cr) as the catalyst without a phase transfer agent because of the mass-diffusion limitations of the liquid-liquid-solid three phase system. After the addition of hexadecyltrimethylammonium bromide as phase transfer agent, the conversion of DBT reached 91.2%, and sulfone was the only product. The conversions of the sulfur compounds were in the order of DBT > 4,6'-DMDBT > BT, which indicates that the electron density of the substrates and steric hindrance may affect the reactivity. The PTA@MIL-101(Cr) could be separated easily with a slight decrease of activity in the consecutive cycles due to the leaching of 9 wt% POM components.

**Figure 13:**
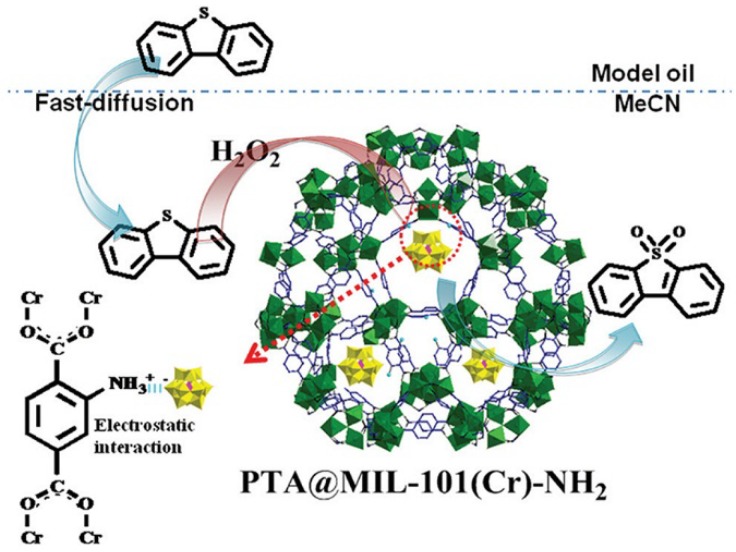
Schematic description of the extractive and catalytic oxidative desulfurization (ECODS) process using PTA@MIL-101(Cr)-NH_2_ as the catalyst. Reproduced with permission from RSC, 2014 [57].

Considering the large window size of MIL-101, it is necessary to enhance the interaction between polyoxoanion and MIL-101 to prevent the POM components from potential leaching. Cao *et al.* prepared PTA@MIL-101(Cr)-NH_2_ catalyst by loading [PW_12_O_40_]^3−^ polyoxoanions on the amino group modified MIL-101(Cr) [57]. The catalytic activity of PTA@MIL-101(Cr)-NH_2_ was evaluated in the extractive and catalytic oxidative desulfurization (ECODS) system as shown in Figure 13. By adding polar DMF or MeCN as the extracting agent, the sulfur compounds could be rapidly transferred from the model oil into the polar phase then oxidized into sulfones. DBT was completely converted to sulfone using MeCN as the polar phase, and the residual DBT in the model oil was negligible. Interestingly, the molar ratio of H_2_O_2_/DBT through the ECODS process in this work was much lower than those in previously reported works. The MIL-101 frameworks showed negligible activity for this reaction but [PW_12_O_40_]^3−^ polyoxoanions had a high catalytic activity with 99.6% conversion which indicates that the catalytic activity of PTA@MIL-101(Cr)-NH_2_ is derived from the [PW_12_O_40_]^3−^ component. The amino groups on the MIL-101 frameworks kept the POM component efficiently from leaching, and the PTA@MIL-101(Cr)-NH_2_ catalyst remained at 99% of activity even after six consecutive cycles.

Moreover, thiophene in fuel may be removed through a photocatalytic reaction. Jiang *et al.* isolated five hybrid POM compounds by reaction of lanthanide ions (La, Ce, Tb, Dy and Er), H_2_pdc (pyridine-2,6-carboxylate) and [BW_12_O_40_]^5−^ under hydrothermal conditions [58]. The lanthanide ions and H_2_pdc molecules connect into 3D rigid Ln^3+^-pdc^2−^ frameworks and the [BW_12_O_40_]^5−^ polyoxoanions locate in the cavities of the structure. The compounds were used as heterogeneous catalysts for photocatalytic degradation of thiophene. Under UV irradiation with O_2_ bubbled into the system, the conversion over the Ce^3+^ containing compound reached 97% at 12 h and CO_2_, SO_3_, and H_2_O were observed as the degradation products.

The synergistic and steric effects of POMs containing HKUST-1 compound were also observed in other catalytic processes. In aerobic oxidation of toxic H_2_S to S_8_, HKUST-1 containing monocopper-substituted [PW_11_CuO_39_]^5−^ polyoxoanions showed significant activity [59]. The structure of the hybrid compound remained stable over a 20 h reaction time and the turnover number of H_2_S was *ca.* 4000. In the controlling experiments, there was no oxidation product when using K_5_[PW_11_CuO_39_] or HKUST-1 solely, and HKUST-1 decomposed rapidly in the reaction solution. In addition, the regioselectivity was clearly observed in the catalytic oxidation of volatile mercaptan to disulfides because the conversion of 2-hydroxyethanethiol was 95%, while the conversion of *p*-toluenethiol was much lower (<30%) due to the window size of HKUST-1 which limited the access of large substrates.

### 4.5. Asymmetric Catalysis

A catalytic asymmetric process for producing enantiomerically enriched compounds is still a big challenge in modern organic synthesis. The commonly explored catalysts for an asymmetric process are small chiral molecules, such as metal complexes or organic molecules. In 2013, Duan and co-workers reported an asymmetric catalytic process over chiral organic–inorganic hybrid POM compounds [60].

**Figure 14:**
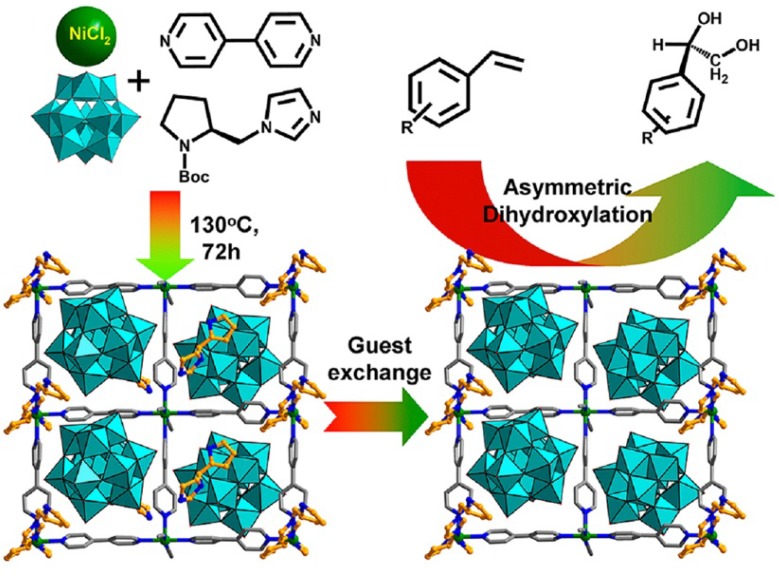
Synthetic procedure of NI-PYI1, showing the guest exchange and the potential amphipathic channel for the asymmetric olefin dihydroxylation. Reproduced with permission from ACS, 2013 [60].

The hybrid POM compounds, namely Ni-PYI, were isolated by the reaction of [BW_12_O_40_]^5−^, Ni ion, 4,4'-bipyridine, and L/D-BCIP (L/D-tert-butoxycarbonyl-2-(imidazole)-1-pyrrolidine). In the structure of Ni-PYI, the 4,4'-bipyridine molecules are connected by Ni ions into 2D layers which are further connected into a 3D rigid framework by L/D-PYI (L/D-pyrrolidin-2-ylimidazole) molecules derived from the decomposition of the L/D-BCIP precursor. The [BW_12_O_40_]^5−^ polyoxoanions and the guest molecules are located in the channels of the framework. Two types of Ni-PYI (L or D type) crystals were isolated as enantiomers due to the configuration of chiral PYI molecules in their structures. The compounds ex-Ni-PYI without guest molecules could be easily obtained by soaking the crystals in dichloromethane solution containing diethylamine. The L-type ex-Ni-PYI compound showed high activity for asymmetric dihydroxylation of styrene with excellent enantioselectivity for (R)-phenyl-1,2-ethanediol (ee > 95%) after 60 h reaction at 313 K. The D-type ex-Ni-PYI exhibited similar catalytic activities but gave products with the opposite chirality as shown in Figure 14.

The catalytic process was also tested over an achiral compound catalyst with high similarity in structure and components composed of Ni ions, 4,4'-bipyridine, and [BW_12_O_40_]^5−^, [Ni_2_H_1.5_(bpy)_5_(H_2_O)_1.5_(Cl)_0.5_][BW_12_O_40_]. The catalytic reaction over this compound did not exhibit any obvious enantioselectivity which indicated that the stereoselectivity in the reactions was dominated by the chiral environment of Ni-PYIs.

The use of ex-Ni-PYI can be extended to other chlorovinylbenzene substrates with comparable activity and asymmetric selectivity. In contrast, when 3,5-di-tert-butyl-4'-vinylbiphenyl with a molecular size larger than the channel was introduced, less than 10% conversion under the same reaction conditions was observed. The results indicated that the reaction occurred indeed in the channel instead of the external surface.

The catalytic reaction over the mixture of Ni_2_H[BW_12_O_40_] and PYI gave a conversion of 45% and an ee value of 15%, which are far lower than those over ex-Ni-PYI as catalyst. The high activity of Ni-PYI is attributed to the synergistic effect of POMs and metal-organic framework components.

In addition to the typical reactions mentioned above, the organic–inorganic hybrid POM compounds have also been applied in other catalytic processes, such as alcoholysis of styrene oxide [61], Knoevenagel condensation [62], dehydration of biomass [63], and some of the works have been summarized [64]. The hybrid POM compounds have shown noteworthy catalytic performance in these reactions.

## 5. Conclusions

A large number of organic–inorganic hybrid POM compounds with bulky or porous structures have been isolated by reaction of POMs, metal ions, and organic ligands under hydrothermal conditions in recent years. As shown above, some of these compounds were studied as heterogeneous catalysts and displayed noticeable performance in various reactions. It should be noted that the organic–inorganic hybrid POM compounds exhibited some attractive features in contrast to the widely studied supported heterogeneous catalysts. The latter catalysts are normally prepared by grafting or impregnation methods, in which the active species are loaded on stable supports, such as clay, oxides, zeolites, and mesoporous materials. Normally, in the liquid–solid reaction system leaching is a major problem in supported catalysts caused by weak interaction between the active species and the supports. The preparation of organic–inorganic hybrid POM compounds have proved to be a unique way of obtaining special functionalized heterogeneous catalysts. All the components are arranged in the structure in high order and structural details such as coordination modes and oxidation states of the metals ions can be determined. The rigid hybrid framework formed by POMs, metal ions, and ligands through a one-step hydrothermal reaction may improve their water resistance due to the widely existing covalent bonds or H-bonds in the structures. The organic–inorganic hybrid POM compounds represent a type of self-supported heterogeneous catalysts with highly tunable structural features of acidity, porosity, charge density, and surface properties. 

The present studies of the organic–inorganic hybrid POM compounds as heterogeneous catalysts show a high catalytic performance, such as in acid and oxidation reactions. However, the research on the catalytic behavior of organic–inorganic hybrid POM compounds is still in its primary stage. In view of the structural feature of POM-based compounds consisting of POMs, metal ions, and ligands, it is expected many potential factors influence their activity, including the type and constituents of the POM unit, the difference in metal centers, the porosity, and the acidity of the compounds. Therefore, the relationship between their catalytic behavior and special structural features may be explored using catalysts with a high structural and compositional similarity. By elimination of potential synergistic effects, it is possible to determine the influence of individual factors on the structure and the catalytic performance. The understanding of catalytic activity demands the accumulation of extensive and thorough researches both in synthetic and catalytic chemistry.
